# The Progressive Spread of the Vascular Wilt Like Pathogen of *Calophyllum* Detected in Ranomafana National Park, Madagascar

**DOI:** 10.3389/ffgc.2020.00091

**Published:** 2020-08-11

**Authors:** Patricia Chapple Wright, Beatriz Otero Jimenez, Paul Rakotonirina, Dina H. Andriananoely, Alexandra Shea, Baovola Ratalata, Jean Claude Razafimahaimodison

**Affiliations:** 1 Department of Anthropology, Stony Brook University, Stony Brook, NY, United States; 2 Centre ValBio Research Campus, Ranomafana, Madagascar; 3 Environmental Studies Program, SUNY New Paltz, New Paltz, NY, United States; 4 Department of Geochemistry and Medicinal Chemistry, University of Fianarantsoa, Fianarantsoa, Madagascar

**Keywords:** *Calophyllum*, Madagascar, Vascular wilt disease, rainforest, Ranomafana National Park, invasive pathogen, tropical conservation

## Abstract

Pathogens are threatening crops worldwide, but little attention has been given to the threat to tree species in undisturbed rainforest. This communication reports the first case of a tree die off caused by a “wilt” in Madagascar. In 2016 while monitoring monthly tree phenology of Ranomafana National Park (RNP), the Centre ValBio research station observed that many *Calophyllum* adult trees had brown wilted leaves. There are three species of *Calophyllum* in this rainforest, *C. paniculatum*, *C. drouhardii*, and *C. milvum*, and all three have contracted this pathogen. Our goal was to document the spead of this suspected wilt in *Calophyllum* trees and determine if site, elevation and DBH had an influence on tree mortality. In 2019 we conducted an inventory of all *Calophyllum* trees in RNP and 42% of the observed trees were dead. The species with the highest mortality was *C. paniculatum*, with 53% of trees dead, followed by *C. milvum* with 18%, and *C. drouhardii* with only 2% of surveyed trees dead. Bark beetle traces were observed in all dead *Calophyllum* trees. Tree death caused by this suspected fungal pathogen has spread across a major river in the area and has been found at mid and high elevations. Our results show that *C. paniculatum* trees with a larger DBH have a higher mortality risk. Our report highlights the importance of fighting invasive pathogens that threaten protected ecosystems.

## INTRODUCTION

Madagascar holds the highest number of endemic families and genera of plants in the world (Myers et al., 2000; Kremen et al., 2008), and based on regional (Ganzhorn et al., 2001) and global estimates (Beech et al., 2017) of tree biodiversity, we estimate that Madagascar holds over 5% of the world’s tree diversity (Dunham et al., 2018). High species diversity, high endemism, and severe threats make Madagascar’s rainforests a focus of intense scientific and conservation interest. While we have an increasing understanding of the role of habitat loss and hunting on Madagascar’s tropical flora and fauna (Brown and Gurevitch, 2004; Mittermeier et al., 2006; Allnutt et al., 2008; Dunham et al., 2008; Irwin et al., 2010; Herrera et al., 2011; Schwitzer et al., 2014), we know little about how invasive disease affects the rainforests of Madagascar. Invasive vertebrates (including the Asian cane toad Marshall et al., 2018; Reardon et al., 2018) and invertebrates (including the marbled crayfish Jones et al., 2009; Gutekunst et al., 2018) have been introduced in the island of Madagascar within the last decade and had a devastating impact on native fauna. There is also evidence that pathogens are transferring from domestic animals (dogs) and invasive rodents (rats) to endemic forest species (lemurs) (Rasambainarivo et al., 2013; Pomerantz et al., 2016; Zohdy et al., 2019).

As with most other tropical plant species, little is known about the ecology and population status of Malagasy forest tree species. For this reason, the emergence of plant pathogens in Madagascar’s rainforest is of particular significance and can lead to undetected disease-induced population declines or extinctions (Anderson et al., 2004; Fisher et al., 2012). The majority of trees in tropical forests are dispersed by vertebrate frugivores (Terborgh et al., 2002) and provide critical resources for supporting vertebrate. In the southeastern rainforest of Madagascar, up to 85% of tree species support the region’s endemic vertebrate frugivores, including birds, bats, and the region’s diverse endemic primates, the lemurs. Loss or declines in tree species population could lead to greater ecosystem effects.

Although the impacts of selective logging precious hardwoods have been documented in Malagasy forests (Irwin et al., 2010; Herrera et al., 2011; Gerber et al., 2012) plant pathogens have not been describes as an important driver of fruit tree decline on the island. Scientific research on pathogens affecting wild plant populations has increased in recent years. This communication seeks to highlight the importance of continuing research in this topic, especially on the effect of invasive pathogens on tropical tree species. Here we present the incidence of the pathological condition on a rainforest population of trees in the family Calophyllaceae. We describe the condition and spread of the disease to *Calophyllum* species within Ranomafana National Park. More specifically, the aim of this study is to examine the effect of elevation, tree size, and location on mortality of *Calophyllum paniculatum* due to the wilt pathogen.

### Tree Wilt Disease

Vascular wilt diseases are caused by pathogenic fungi, bacteria or nematodes that enter the water-conducting xylem vessels of a plant, then proliferate within the vessels, causing water blockage (Tattar, 2012). Symptoms, including the *Calophyllum* wilt described here, include wilting and death of the leaves, followed often by death or serious impairment of the whole plant (Ploetz et al., 2011). As a group, the vascular wilts are among the most devastating plant diseases (Ploetz et al., 2011). Insects are one of the prominent invasive species groups worldwide (Biedermann et al., 2019). Bark beetles are one of the main dispersers of vascular wilt causing agents such as fungi and nematodes, and have devastated forests in North America, and Europe (Kudela et al., 1976; Wainhouse et al., 1998; Webber et al., 1999). However, most attention is given to plants that are used by humans as food crops (Mayfield et al., 2008) such as the avocado and mango, and not forest tree species (Ploetz et al., 2011; Rodgers et al., 2014; da Silva Galdino et al., 2016).

A vascular wild disease was detected in *Calophyllum inophyllum* trees on the island of Mauritius in 1939 just 1132 km from Madagascar (Webber et al., 1999). In 1994 on the island of Mahe on the Seychelles, 1834 km from Madagascar, the presence of the wilt was described affecting *Calophyllum* trees, and it was hypothesized that it was being spread by the bark beetle *Cryphalus trypanus* (Wainhouse et al., 1998; Webber et al., 1999). Additionally, this disease has been reported in other tropical regions such as, El Salvador and Cuba (Kudela et al., 1976). However, this is the first time that a wilt pathogen has been reported in Madagascar. Based on these studies and our field observations we suspect that the cause of the wilt disease in *Calophyllum* trees in RNP is the fungal pathogen dispersed by the bark beetles. However, our investigation of the cause of the wilt is ongoing.

### *Calophyllum* Value

*Calophyllum* trees can grow up to 30 meters in height and 90 DBH. *Calophyllum* wood is used to build boats, for construction, carpentry, flooring, furniture, cabinet work, and musical instruments (Damon, 2016). This tree is known for its chemistry with a variety of secondary metabolites isolated such as coumarins, xanthones, flavonoids and triterpenes which have cytotoxic, anti-HIV and antimicrobial properties (Vandana, 2017). Used as a medicinal plant by local healers, *Calophyllum* can treat peptic ulcers, tumors, infections, pain, and inflammation (Ratalata, 2007). In addition, *Calophyllum* trees have been reported as an important source of food for frugivorous birds (Pratt, 1982; Galetti, 1993), bats (Mello et al., 2005), and lemurs (Birkinshaw, 2001; Martinez and Razafindratsima, 2014).

## MATERIALS AND METHODS

### Study Site

**FIGURE 1 ∣:**
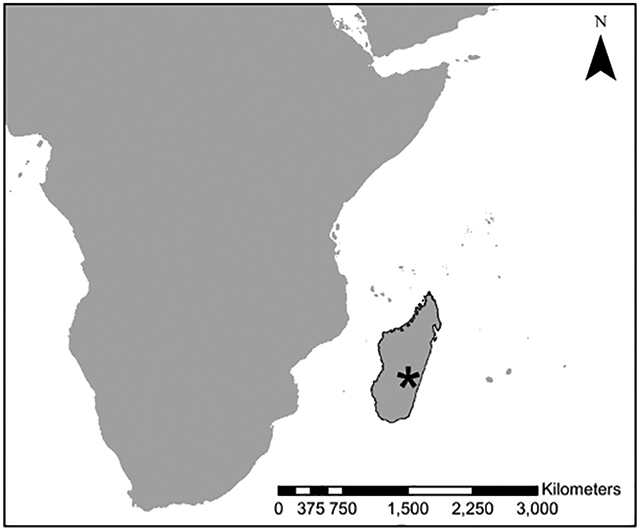
Map of Madagascar showing the location of Ranomafana National Park as an asterisk.

Our study was conducted in Ranomafana National Park located in the southeastern rainforest belt of Madagascar (47°18′- 47°37′E, 21°02′- 21°25′S) and home to Centre ValBio research station (Wright et al., 2012; Figure 1). The park is comprised of 41,600 ha of evergreen montane rainforest, ranging in elevation from 600 to 1480 m (Wright and Andriamihaja, 2002). The park hosts over 330 tree species (Razafindratsima and Dunham, 2015), 85% of which are dispersed by frugivores (Razafindratsima and Dunham, 2016). The Centre ValBio tree phenology team has been recording fruiting phenology data since 1987 for 71 common tree species (representing 24 families and 46 genera). Trees are monitored for fruiting and flowering once a month (Wright et al., 2012). During the May 2016 monthly monitoring, the Centre ValBio Botanical team reported that *C. paniculatum* was dying. This discovery spearheaded an active search for *Calophyllum* trees in Ranomafana National Park.

### *Calophyllum* Census

In 2016 the first diseased *C. paniculatum* tree was detected by observation of dried and dead leaves during monthly phenological monitoring. In 2019 three Centre ValBio botanists systematically searched on trail for the *Calophyllum* trees in 5 sites within Ranomafana National Park. We surveyed each site 8 h every day for 6 days covering an area of approximately 3 km^2^ at each site. Our efforts resulted in the identification of more than 1,000 *Calophyllum* trees of all three species. For each *Calophyllum* tree the DBH, elevation, GPS coordinates, and tree condition (i.e., dead by disease, dead, or alive) were recorded. Trees were considered dead by disease if the presence of beetle tracks in the trunk and wilted leaves was observed. We estimated the effect of elevation, site, and DBH on tree survival through a binomial generalized linear model in R.

## RESULTS

**FIGURE 2 ∣:**
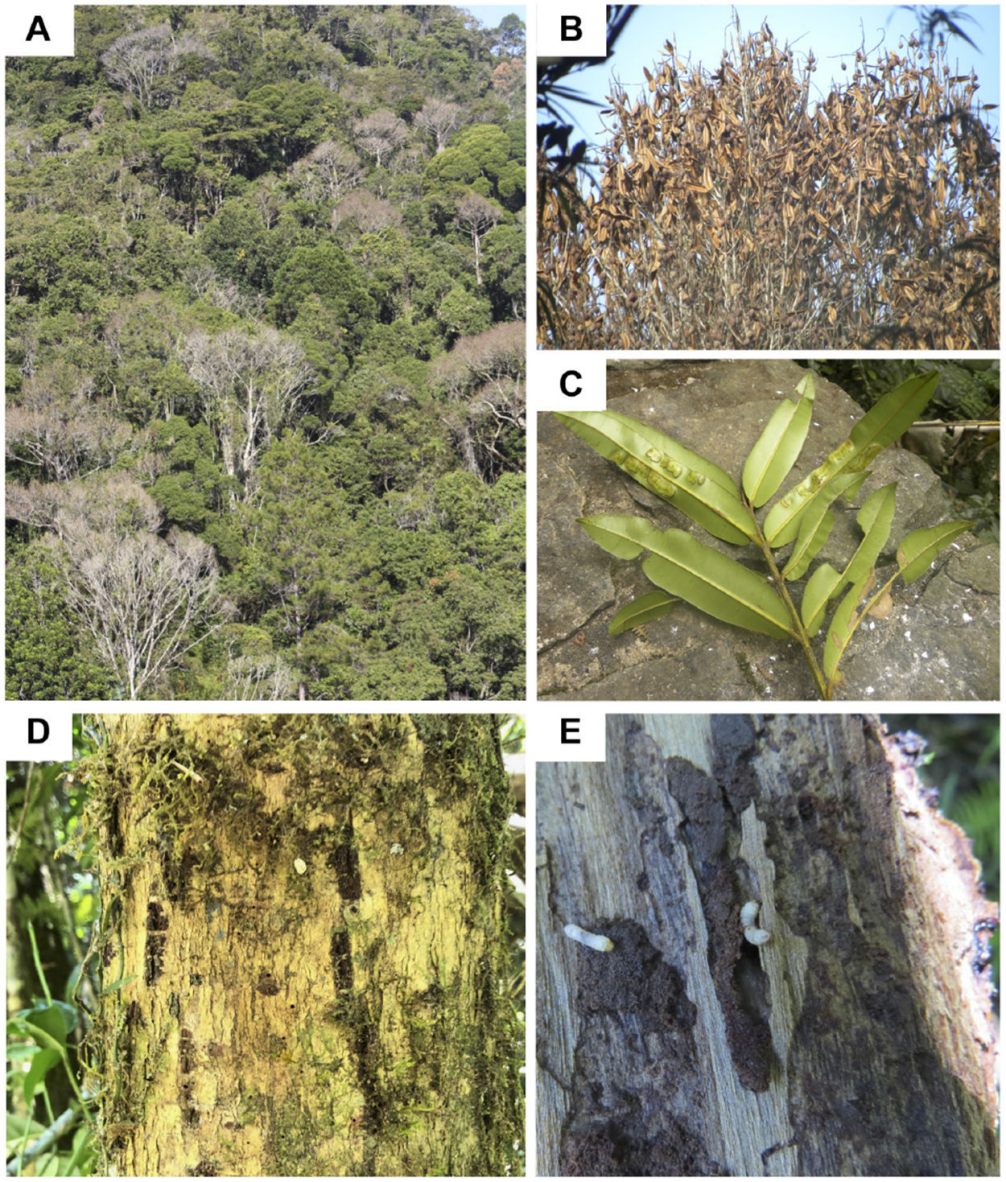
Photos of *Calophyllum* trees in Ranomafana National Park, **(A)** landscape of forest with dead trees within the park, **(B)** closeup of tree with dead leaves, **(C)** close up of the diseased leaves, **(D)** the bark of a dead *C. paniculatum* tree with grooves perhaps caused by bark beetles, and **(E)** suspected bark beetle larvae in *Calophyllum* tree trunk.

**FIGURE 3 ∣:**
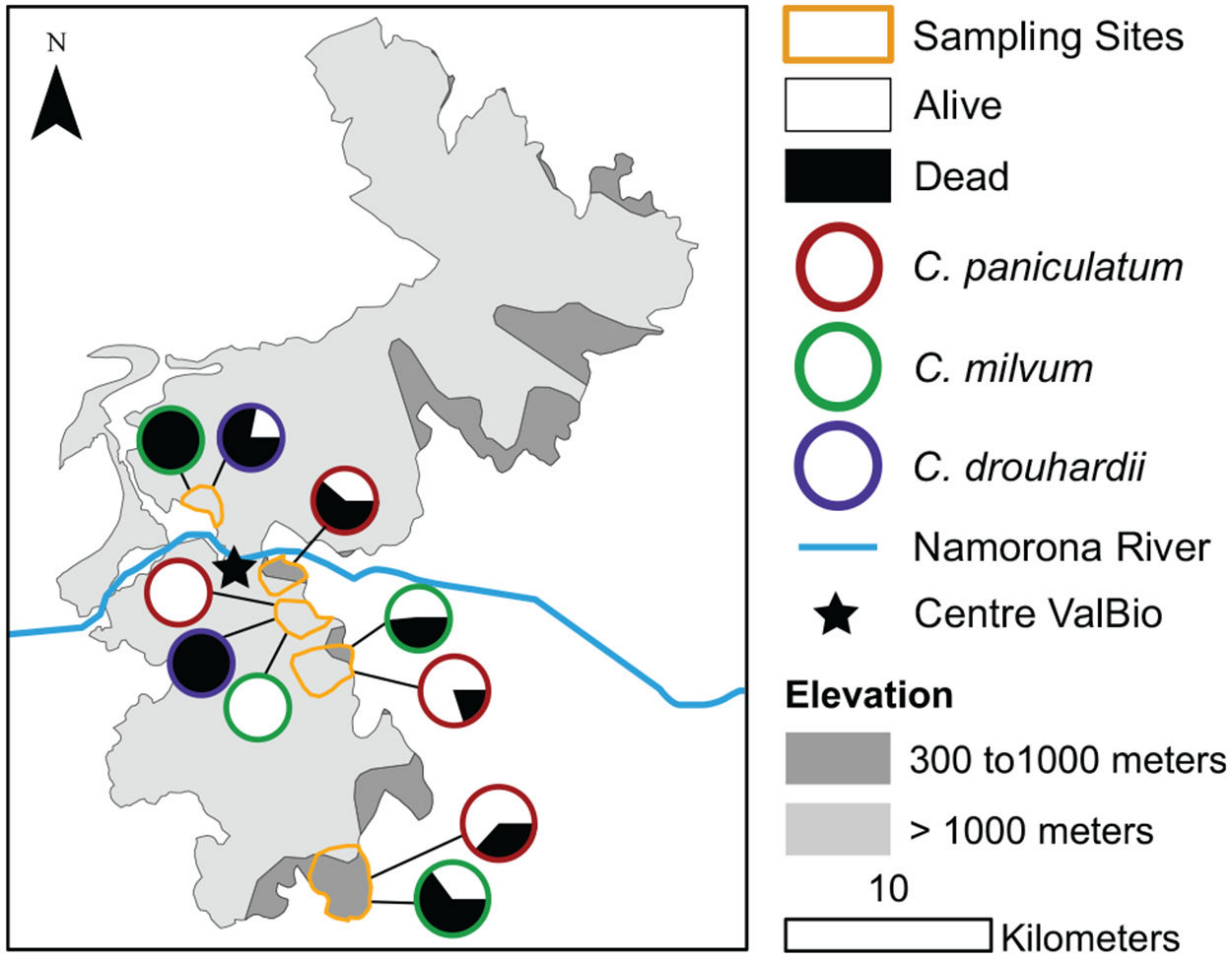
Map of surveys of *Calophyllum spp*. within five sites in Ranomafana National Park, Madagascar with elevation and proportion of dead and living trees by species.

**FIGURE 4 ∣:**
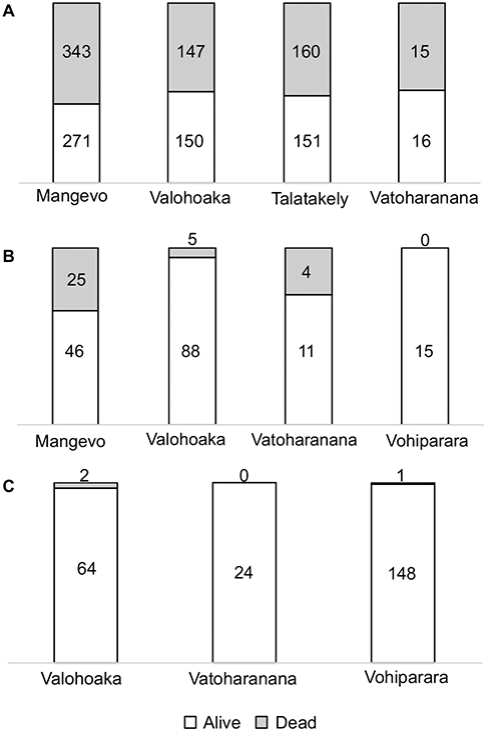
Proportion of dead (gray) and alive (white) trees per species of *Calophyllum* per site surveyed: **(A)** *Calophyllum paniculatum*, **(B)** *Calophyllum milvum*, and **(C)** *Calophyllum drouhardii*. Numbers within column represent the number of trees in each category.

**FIGURE 5 ∣:**
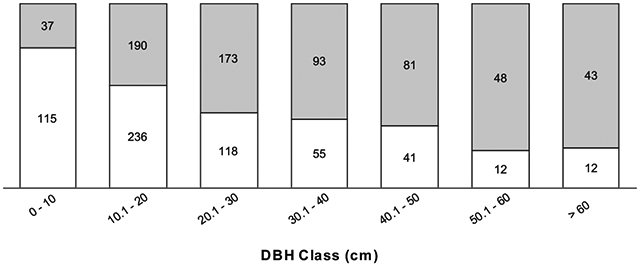
Proportion of dead (gray) vs. alive (white) trees per DBH class of *Calophyllum paniculatum* in Ranomafana National Park. Numbers within columns indicate the number of individual trees in each category (dead or alive).

The disease progresses from a few leaves with welts, which turn brown, and die a few branches at a time (Figures 2A-C). More branches become progressively brown and the trunk is covered with vertical tracks (Figure 2D) and larvae (Figure 2E) that appear to be evidence of the bark beetle. The dead trees within the forest landscape show the dispersion of the *Calophyllum* trees (Figure 2A). In 2019 the disease was widespread in *Calophyllum* species and death by disease was detected in all 5 sites surveyed (Figure 3). All observed dead trees had evidence of contracting the pathogen and represented 42% (*n* = 702) of all the trees surveyed. The species with the highest mortality was *C. paniculatum*, with 53% of trees dead, followed by *C. milvum* with 18%, and *C. drouhardii* with only 2% of surveyed trees dead. Above 1000 m elevation, there were few *C. paniculatum* trees, and they are replaced by other species in this genus, *C. drouhardii* and *C. milvum* (Figure 3). Tree mortality varied by location and species (Figure 4). However, statistical analysis were only conducted for the most widespread species, *C. paniculatum*. Results from the generalized linear model (GLM) show that *C. paniculatum* trees of larger DBH are more likely to contract the disease and die (Table 1 and Figure 5). Elevation was not a significant variable in predicting tree survival (Table 1). Additionally, based on the GLM *C. paniculatum* trees present in the Talatakely site had a higher probability of dying from the disease than those in other sites (Table 1). During the time of the study bark beetles were never observed, but the vertical tracks implying bark beetle infestation were obvious on trunks of dead and dying trees, and larvae were observed (Figures 2D,E).

## DISCUSSION

Understanding how rapidly and how far disease epidemics can spread through species-rich tropical forests should be a priority for conservation planning (Gilbert and Hubbell, 1996; Anderson et al., 2004). Yet basic data on invasive tree pathogens in tropical forests is lacking. In this short communication we describe the first incidence of the pathological condition of wilt in Madagascar on a rainforest population of *Calophyllum* trees in the Ranomafana National Park.

In 2016 for the first time in the eastern rainforests of Madagascar a pathogen was detected to attack a primary rainforest tree. In this Malagasy case, the susceptibility of the trees may be caused by drought stress, and climate change might be implicated as dry seasons have become protracted in the last decade (Parmesan and Yohe, 2003; Ingram and Dawson, 2005; Wright, 2006; Hannah et al., 2008; Moritz and Agudo, 2013; Brown et al., 2015).

Our results highlighted the influence of tree DBH on tree survival to the wilt disease. We found that *C. paniculatum* with larger DBH were more likely to contract and die from the wilt disease. These results support patterns found on pine wilt disease in South Korea pine forests where researchers found higher risk rate of contracting the disease in pines with a DBH > 10 cm (Park et al., 2013). This pattern could be because trees with higher DBH have a larger canopy area and will be easier for beetles, the potential wilt fungi dispersers, to find these trees. We also found that *C. paniculatum* trees located in Talatekely site had a higher probability of contracting and dying due to the disease. The Talatekely site is closer to the entrance of the national park and has a higher number of visitors than other sites, increasing the potential of plant pathogen dispersal from outside the park.

We will need a census of pathogens involved tree symptoms in Madagascar and further work is needed to demonstrate Koch postulate.

Conservation biology is perhaps one of the most interdisciplinary in the biological sciences, but plant pathology has played only a peripheral role (Gilbert and Hubbell, 1996). The probability that introduced diseases will invade, spread and kill in protected tropical ecosystems is increasing with climate change and fighting these new pathogens should be a priority in conservation biology (Gilbert and Hubbell, 1996; Roy et al., 2017).

## Figures and Tables

**TABLE 1 ∣:** Parameter estimates of general linear model for *Calophyllum paniculatum* survival including interaction term between sampling sites and DBH.

Variable	Estimate	Standard error	*p*-value
(Intercept)	**0.87**	**0.18**	**<0.001**
DBH	**−0.04**	**0.01**	**<0.001**
Site-Talatakely	**1.68**	**0.37**	**<0.001**
Site-Valohoaka	0.22	0.33	0.506
Site-Vatoharanana	1.47	1.06	0.167
DBH X Site-Talatakely	**−0.15**	**0.02**	**<0.001**
DBH X Site-Valohoaka	0.00	0.01	0.808
DBH X Site-Vatoharanana	**−0.02**	0.03	0.381

Bold values represent statistically significant results (p-values > 0.5).

## Data Availability

The datasets generated for this study are available on request to the corresponding author.
